# A Complexed Initiating System AlCl_3_·Phenetole/TiCl_4_·H_2_O with Dominant Synergistic Effect for Efficient Synthesis of High Molecular Weight Polyisobutylene

**DOI:** 10.3390/polym11122121

**Published:** 2019-12-17

**Authors:** Yulong Jin, Liang Chen, Xing Guo, Linfeng Xu, Zhihua Zhu, Zhen Liu, Ruihua Cheng, Boping Liu

**Affiliations:** 1 College of Materials and Energy, South China Agricultural University, Wushan Road 483, Guangzhou 510630, China; jyl@scau.edu.cn; 2 State Key Laboratory of Chemical Engineering, East China University of Science and Technology, Meilong Road 130, Shanghai 200237, China; liangchenwk@126.com (L.C.); ecustguoxing@163.com (X.G.); xulingfeng1026@126.com (L.X.); zhhzhu@ecust.edu.cn (Z.Z.); liuzhen@ecust.edu.cn (Z.L.); rhcheng@ecust.edu.cn (R.C.)

**Keywords:** cationic polymerization, isobutylene, complexed catalyst, high molecular weight, synergistic effect

## Abstract

A complexed initiating system AlCl_3_·phenetole/TiCl_4_·H_2_O was prepared by simply compounding AlCl_3_/phenetole and TiCl_4_/H_2_O and used for cationic polymerization of isobutylene. It was found AlCl_3_·phenetole/TiCl_4_·H_2_O exhibited activities 1.2–3 times higher than those of AlCl_3_/phenetole, and more than an order of magnitude higher than those of TiCl_4_/H_2_O, which indicated a notable synergistic effect produced in the complexed system. In addition, due to the higher activity of AlCl_3_·phenetole/TiCl_4_·H_2_O, lower coinitiator concentration and polymerization temperature, as well as higher monomer concentration were more favored for this complexed initiating system to produce polyisobutylene (PIB) with reasonable molecular weight (M_w_) and molecular weight distribution (MWD). Furthermore, high molecular weight polyisobutylene (HPIB) with M_w_ = 1–3 × 10^5^ g·mol^−1^ could be successfully produced by the complexed catalyst system at T_p_ = −60 to −40 °C. As a whole, the high activity as well as the simple preparation procedures of the complexed initiating system offer us a unique approach for the production of HPIB with improved efficiency.

## 1. Introduction

High molecular weight polyisobutylene (HPIB), which owns viscosity average molecular weight (M_v_) higher than 10^5^ g·mol^−1^, is one of the most unusual polymers and exhibits numerous excellent properties such as extremely low gas permeability, outstanding thermal stability and low fragility [1,2]. Thus it has been applied in the manufacture of sealant, automotive, medical equipment and so forth [3]. Commercially, HPIB is produced with Lewis acid based initiating systems through the cationic polymerization of isobutylene (IB), and polymerization temperature (T_p_) as low as −100 °C is necessary to depress the chain transfer or termination reaction and achieve high molecular weight (M_w_) polymers [4,5,6]. However, it is obvious that such a low T_p_ is critical to both energy and equipment costs. Therefore, developing novel initiating systems and manufacture processes for the synthesis of HPIB at elevated T_p_ is significant.

The novel organometallic catalysts were reported to have an advantage over the synthesis of HPIB [7]. For examples, in the presence of B(C_6_F_5_)_3_ and zirconocenes, Bochmann et al. found polyisobutylene (PIB) with M_w_ higher than 10^6^ g·mol^−1^ could be obtained at T_p_ closed to −70 °C [8]. Jörg et al. reported a dicationic zirconocene for the synthesis of HPIB with M_w_ higher than 3 × 10^5^ g·mol^−1^ at T_p_ below −50 °C [9]. Baird et al. reported that HPIB with M_w_ =1–6 × 10^5^ g·mol^−1^ could be produced at T_p_ = −50 to −10 °C by a half-titanocene coordinated with B(C_6_F_5_)_3_ [10]. It is generally recognized that the weakly coordinating anions (WCAs) such as B(C_6_F_5_)_3_ is indispensable for the organometallic catalysts, as the WCAs act as stabilizer to the active sites and retard the chain transfer reaction [11]. However, much attention has still been paid on the conventional Lewis acid systems both in the academic and industrial fields, as the synthetic routes for these organometallic catalysts are much more complicated, and the cost is also relatively higher. [12]. On the other hand, HPIB could also be produced at elevated T_p_ with Lewis acid initiating systems, if proper reaction conditions are chosen. Particularly, the AlCl_3_-based initiating systems, which are widely investigated in both academy and industry for the production of PIB, butyl rubber and other cationic polymers, are among the most favored candidates for the synthesis of HPIB because of the low price, low dosage and high activity [3,13,14,15,16,17,18,19,20]. Lu and coworkers took advantage of the microflow reaction system in perfect mixing and heat transfer performances, as well as narrow residence time distribution, thus a reaction system with enhanced homogeneity and controllability could be created, and PIB with weight-average molecular weight (M_w_) higher than 1 × 10^5^ g·mol^−1^ was produced by AlCl_3_/H_2_O at T_p_ = −30 to −10 °C [21]. Csihony et al. reported a novel initiating system of Lewis acid anion Al_2_Cl_7_^−^ trapped in micelles consisting of functionalized low molecular weight PIB. The activity of the system was high enough that HPIB with M_w_ = 1.7–9 × 10^5^ g·mol^−1^ and molecular weight distribution (MWD) = 13–47 could be produced at T_p_ = −76 °C [2]. Wu et al. prepared a series of AlCl_3_/H_2_O/ED (ED = electron donor = methyl benzoate, ethyl benzoate, and methyl acrylate) initiating systems. It was found that in the presence of EDs, the M_w_ of the PIB could reach to 6–8 × 10^5^ g·mol^−1^ at T_p_= −80 to −70 °C, which was even higher than that produced by AlCl_3_/H_2_O at T_p_ = −100 °C [3,22]. Later on, the same group reported another novel initiating system of AlCl_3_/H_2_O/veratrole, and HPIB with M_w_ higher than 1 × 10^6^ g·mol^−1^ could be synthesized at T_p_ = −80 °C. It was argued that the EDs were able to interact with the active centers and affect the nucleophilicity and polarity of the microsurroundings around the active centers. As a consequence, the cationic polymerization proceeded in a more controllable way, and side reactions such as chain transfer and termination were depressed, but the propagation rate mostly declined with the increased concentration of EDs [23]. Kostjuk and coworkers found H_2_O/iBu_2_AlCl/toluene was able to afford PIB with high M_w_ at T_p_ = −20 °C because of the weak basicity of toluene, which would help to stabilize the active species. While for iBuAlCl_2_ with stronger Lewis acidity, additional ether was needed to suppress side reactions and obtain HPIBs [24]. The same group also disclosed that alkoxy aluminum chlorides-based systems H_2_O/(RO)_0.8+*n*_AlCl_2.2−*n*_/*n*-hexane (R = Bu, Hex or iPr; *n* = 0–0.4) could produce PIBs with low to medium M_w_ and relatively narrower MWD. It was found the oxygen in the coinitiator played a key role as electron donor to stabilize the active species, which would retard the isomerization of the macrocation and chain scission and benefit the synthesis of high M_w_ polymers. Therefore, PIBs with M_w_ up to 1.2 × 10^5^ g·mol^−1^ could be produced at elevated T_p_ = −20 to 20 °C [25].

Recently, an endeavor was made in our group to make HPIB with AlCl_3_/ROH (R = H, Me, Et, Bu, ^t^Bu and Ph) or AlCl_3_/ether (ether = diethyl ether, butyl ether, anisole and phenetole) initiating systems as well, and HPIB with M_w_ > 1 × 10^5^ g·mol^−1^ could be generally produced at relatively elevated T_p_ = −60 °C. Particularly, AlCl_3_/phenetole showed the highest efficiency for the synthesis of HPIB among these systems [26]. More recently, a novel complexed system consisting of BF_3_·EtOH/TiCl_4_·H_2_O was reported in our group, and remarkable synergistic effect in its catalytic efficiency could be observed due to this complexation [27]. However, it should be noted that BF_3_ is highly toxic and environmental unfriendly. Therefore, in this contribution, we tried to make use of AlCl_3_, which is relatively greener and more economical than BF_3_, to give another complexed initiating system AlCl_3_·phenetole/TiCl_4_·H_2_O, aiming to produce HPIB with improved efficiency.

## 2. Materials and Methods 

### 2.1. Raw Material

Isobutylene (Wetry Standard Gas (Shanghai) Co., Ltd., 99.80%, Shanghai, China), anhydrous AlCl_3_ (Shanghai Aladdin Bio-Chem Technology Co., LTD, 99%, Shanghai, China), TiCl_4_ (Lingfeng Chemical Co., Ltd., 99%, Shanghai, China) and Phenetole (Shanghai Aladdin Bio-Chem Technology Co., LTD, 99%, Shanghai, China) were used as received. CH_2_Cl_2_ (Lingfeng Chemical Co., Ltd., 99%, Shanghai, China) was distilled over CaH_2_ under the atmosphere of N_2_ for more than 6 h before use. N_2_ (Wetry Standard Gas (Shanghai) Co., Ltd., 99.999%, Shanghai, China) was further purified by passing through two columns packed with 4A and silver molecular sieves, respectively. 

### 2.2. Catalyst Preparation and Polymerization

All the polymerizations were implemented in three-necked flasks (ca. 250 mL) under the atmosphere of N_2_. Standard Schlenk technique was applied to avoid the introduction of air into the reaction system. The isobutylene (IB) gas was firstly liquefied by being introduced to a three-necked flask prechilled in a cooler at the target T_p_, and a certain amount of CH_2_Cl_2_ was transferred to the flask by a syringe to get the monomer solution. Afterwards, in a glove box under N_2_ atmosphere, a certain amount of AlCl_3_ powder was weighted and sealed in a glass tube. To make the solution of the complexed catalyst, the powder was introduced to another three-necked flask and flushed by CH_2_Cl_2_, then phenetole, TiCl_4_ and H_2_O were sequentially injected into the flask by syringes. Subsequently, both the monomer and catalyst solutions were kept at T_p_ for at least half an hour. To start polymerization, the catalyst solution was transferred to the monomer solution, and then the reaction system was magnetically stirred and kept for a scheduled time. Subsequently, about 2 mL NaOH/ethanol mixture was poured into the reactor to terminate the polymerization process. The quenched mixture was separated from the solvent by vacuum filtration and washed by deionized water and EtOH three times, respectively. Afterwards, it was dried in vacuum at 40 °C overnight, and the product was attained and weighted. Activities = m_(PIB)_/(n_cat_·t) were calculated to compare the efficiencies of these catalysts, where m_(PIB)_ was the weight of the obtained polymers in kilograms, n_cat_ was the amount of the added AlCl_3_ and TiCl_4_ in molar number, and t was the reaction time in hour.

### 2.3. Polymer Characterization

The weight average molecular weight (M_w_) and MWD (M_w_/M_n_) of the obtained PIBs were characterized by gel permeation chromatography (GPC, Waters-1515) combined with two Mixed-C columns. Typically, 10 mg PIB was dissolved in 10 mL tetrahydrofuran (THF) to make polymer solutions with a concentration of 1mg·mL^−1^, which was then measured at 35 °C at a flow rate of 1.0 mL·min^−1^. The columns were calibrated by polystyrene standards with narrow MWD.

## 3. Results and Discussion 

### 3.1. Effect of Coinitiator Concentration

The effect of coinitiator concentration on the polymerization behaviors of reference and complexed catalysts was investigated, and the results were listed in Table 1. At coinitiator concentration lower than 2.5 mmol·L^−1^, it was obviously for both catalysts that the monomer conversions were enhanced with the increasing catalyst concentration, but to achieve a parallel conversion, lower catalyst concentration was needed for the complexed one, which implied the higher efficiency of the complexed catalyst. It could also be directly reflected by the Δ value in Table 1, showing that the activities of AlCl_3_·phenetole/TiCl_4_·H_2_O were 2–3 times higher than those of AlCl_3_/phenetole at identical reaction conditions. When the concentration of coinitiator was lower than 1 mmol·L^−1^, no polymer could be detected for the AlCl_3_/phenetole system, probably due to the comparable trace concentration of impurity to that of the active sites [28]. However, for the complexed catalyst, a monomer conversion higher than 30% could still be obtained at this low coinitiator concentration. With respect to polymer products, monomodal HPIB with M_w_ higher than 2 × 10^5^ g·mol^−1^ and MWD = 2–4 could be produced with both catalysts at low concentration, but the complexed catalyst system was apt to produce PIB with lower M_w_ and broader MWD when compared with AlCl_3_/phenetole, as the high polymerization activity made the process control more difficult. When the concentration of coinitiator further increased to higher than 2.5 mmol·L^−1^, the efficiencies between the two catalyst systems were indistinct. 

**Figure 1:**
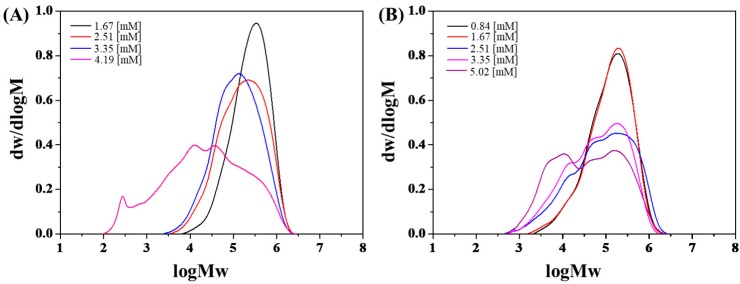
The gel permeation chromatography (GPC) curves of the PIB produced by (**A**) AlCl_3_/phenetole and (**B**) AlCl_3_·phenetole /TiCl_4_·H_2_O at various [AlCl_3_ + TiCl_4_] concentrations. The other reaction conditions are listed in Table 1.

**Figure 2:**
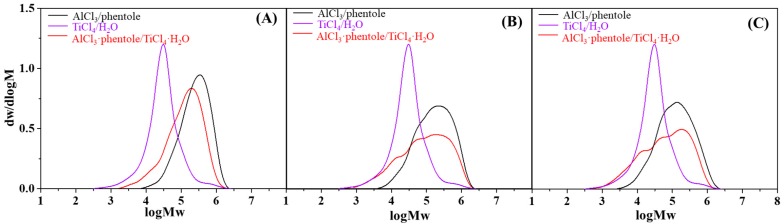
The GPC curves of PIB produced by AlCl_3_/phenetole, TiCl_4_/H_2_O and AlCl_3_·phenetole/TiCl_4_·H_2_O, (**A**) the GPC curves of PIB produced from run 3, 4 and 11 in Table 1; (**B**) the GPC curves of PIB produced from run 5, 6 and 11 in Table 1 and (**C**) the GPC curves of PIB produced from run 7, 8 and 11 in Table 1.

In addition, the M_w_ of the PIB decreased and multimodal MWD could be observed (see Figure 1). It was likely that active species with distinct kinetic characteristics existed at high complex concentration. When looking into the GPC curves about the polymers produced by the catalysts before and after complexation together (see Figure 2), it was found the curve of the PIB made by AlCl_3_·phenetole/TiCl_4_·H_2_O was analogous to that by AlCl_3_/phenetole at low catalyst concentration. While it tended to be the combination of those by AlCl_3_/phenetole and TiCl_4_/H_2_O at high catalyst concentration. However, it was not the result of separate working of the two reference catalysts. Since such a situation would bring about bimodal but not multimodal MWD, and the activities of the complexed catalyst were also difficult to get close to or even higher than those of AlCl_3_/phenetole, if we consider the much lower efficiency of TiCl_4_/H_2_O (see Figures S1–S4 in Supporting Information). Another possibility for the decreased M_w_ and broadened MWD was presumably owing to the monomer starvation, which would lead to intensified side reactions like chain transfer and termination [29]. Moreover, chain scission should be taken into account as well, because it got more importantly under monomer starvation and was reported to be frequent in the AlCl_3_-based system for cationic polymerization [15,30]. This could also be directly reflected by the severely decreased M_n_ when higher catalyst concentration was used. It indicated that low coinitiator concentration is more favored for both initiating systems, specifically for AlCl_3_·phenetole/TiCl_4_·H_2_O because of its higher activity. It preliminarily indicated that an obvious synergistic effect was also produced in the complexed catalyst as that discovered in BF_3_·EtOH/TiCl_4_·H_2_O [27]. To further ensure this synergy, several control experiments with initiating systems consisting of two or three components were also investigated (see Run 11–16 in Table 1). It could be seen that the three components catalysts, as well as 1TiCl_4_·1phenetole showed very low or even no activities, while 1TiCl_4_·1H_2_O and 1AlCl_3_·1H_2_O exhibited moderate activities of less than 5 kg PIB·mol^−1^TiCl_4_·h^−1^ and 80 kg PIB·mol^−1^AlCl_3_·h^−1^, respectively. However, under the similar reaction conditions, 1AlCl_3_·1phenetole/1TiCl_4_·1H_2_O gave activities of more than 130 kg PIB·mol^−1^(AlCl_3_+TiCl_4_)·h^−1^ and presented an obvious synergistic effect. Such a synergistic effect is very interesting, but was difficult to be illustrated at present. Marek and coworkers also found a similar synergistic effect in mixture consisting of two types of Lewis acids for IB polymerization in the absence of initiators, and it was proposed to result from the formation of very active ion pair due to the inter-ionization between the two Lewis acids with different acidity. However, as a certain amount of H_2_O and phenetole was added in our case, making the existence of a large amount of free Lewis acid unlikely, thus the inter-ionization mechanism was almost impossible. In addition, this synergistic effect could also originate from the modification of the counterion by TiCl_4_, improving the stability of the growing species for IB insertion [31,32,33]. Nevertheless, deeper investigation is still needed to uncover the mechanism behind.

### 3.2. Effect of Reaction Temperature 

**Figure 3:**
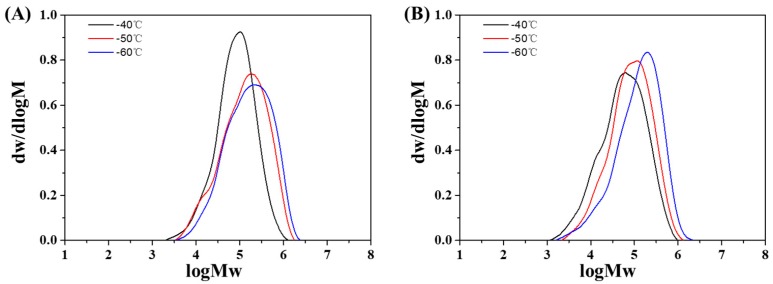
The GPC curves of the PIB produced by (**A**) AlCl_3_/phenetole and (**B**) AlCl_3_·phenetole/ TiCl_4_·H_2_O at different T_p_. The other reaction conditions are listed in Table 2.

Reaction temperature (T_p_) is one of the most important factors in the regulation of catalysis behaviors for cationic polymerization. Thus the effect of reaction temperature was also investigated at T_p_ = −40 to −60 °C commonly used for the synthesis of PIB (see Table 2). Primarily, it could be seen that the activities of the complexed catalyst were about 1.5–3 times higher than those of the uncomplexed ones under the investigated T_p_. Additionally, it was conspicuous that both the monomer conversion and activities of the catalysts went up with increasing T_p_, which were contrary to the results mostly reported for cationic polymerization that active sites collapsed more easily at higher T_p_, and the conversion was kept almost the same or turned down consequently [5,13,34,35]. However, such a deviation was also disclosed elsewhere [9,28]. It was proposed the tightness of the initiator/coinitiator complex got strengthened at lower T_p_. Consequently, the concentration of the free Lewis acids, which play a role as coinitiator for cationic polymerization got lower as well [36]. It could also be partially attributed to the faster generation of active sites in comparison to their decay at higher T_p_ for both initiating systems. In addition, the viscosity of the reaction system got higher at lower T_p_, and it could be more severe in the system for HPIB production, as gel-like PIB with relatively high M_w_ was generally produced and suspended in the solvent. This would inhibit the smooth going of heat and mass transfer processes, and also improve the possibility of the mechanical occlusion of catalysts by polymer and impair the efficiency of the catalysts [21]. While high T_p_ would help to create more homogeneous reaction conditions by improving the dissolubility of the polymer. Pertaining to the produced HPIB, the M_w_ decreased monotonously with increasing T_p_, as the chain transfer reaction is more sensitive to temperature changes than chain propagation. The MWD of the HPIB also got slightly narrower at higher T_p_. It is most likely that the initiation and chain transfer processes became more competent, while the apparent rate constant for chain propagation was kept almost the same at higher T_p_ [37,38]. In addition, the more homogeneous reaction conditions at increasing T_p_ should be taken into account as well. However, an exception was seen at run No 3 and 6 in Table 2, where an increase in MWD was observed. This could be possibly caused by chain scission during polymerization, which was exhibited by the additional low M_w_ tail in the GPC curves of the polymers (see Figure 3). In comparison to AlCl_3_/phenetole, the complexed catalyst still tended to produce HPIB with lower M_w_ and broader MWD, which indicated more dominant side reactions such as chain transfer, termination and scission in the latter initiating system. Moreover, with increasing T_p_, a smaller difference in activities between the two systems could be observed, indicating the active sites in the complexed one were more sensitive to temperature and more frequently terminated at higher T_p_. It implies the synergistic effect demonstrated in the complexed system not only improves the catalytic efficiency greatly but also poses a challenge to the process controllability of the polymerization reaction. Therefore, lower T_p_ seemed to be more crucial to AlCl_3_·phenetole/TiCl_4_·H_2_O than AlCl_3_/phenetole. However, as a whole, HPIB with M_w_ = 1–2.8 × 10^5^ g·mol^−1^ and MWD = 2.8–4.1 could be produced with both initiating systems.

### 3.3. Effect of Monomer Concentration

High monomer concentration ([IB]) is always desired in industry to save the cost, if high conversion could be achieved at the same time. The effect of [IB] on the polymerization behaviors of both initiating systems is shown in Table 3. With regarding to AlCl_3_/phenetole, the monomer conversion decreased from 70% to 15% when [IB] increased from 2.4 to 5.1 mol·L^−1^, and the activity also followed the same trend. This decline was possibly derived from the decreasing concentration of the polar solvent CH_2_Cl_2_ caused by the increasing [IB], as an active ion pair in AlCl_3_/phenetole were more likely generated in more polar conditions (see Figure S5). For the complexed catalyst, the monomer conversion was kept at about 90% when [IB] increased from 2.4 to 4 mol·L^−1^, but obvious drop could also be observed when [IB] increased further. It implies a wider range of applicable [IB] for AlCl_3_/phenetole/TiCl_4_·H_2_O. In addition, the activities of the complexed one were still kept 1.2–2.3 times higher than those of the uncomplexed one. With respect to the produced polymers, the M_w_ increased with the increasing [IB], as chain propagation was more favored than transfer at higher [IB], and HPIB with M_w_ = 1.5–3 × 10^5^ g·mol^−1^ could be generally produced. However, when compared with AlCl_3_/phenetole, the complexed catalyst was more likely to produce polymer with lower M_w_ and much broader MWD at the same reaction conditions, and this trend was more distinct at lower [IB]. Again, it is possibly due to the monomer starved condition met in the complexed system, as the high monomer conversion at low [IB] would result in more serious chain scission, which could bring about lowered M_w_ and broadened MWD [15]. Meanwhile, the polarity of the reaction environment was enhanced at lower [IB], which would facilitate the generation of active sites with stronger cationicity and result in more intensified side reactions [35]. 

### 3.4. Effect of Polymerization Time

The polymerization behaviors of the catalysts are likely to change with polymerization time (t_p_), as the composition of the reaction system is very complex and would vary with time as well. Thus t_p_ in the range of 1–30 min was investigated, and the results are given in Table 4. It is evident that the monomer conversion got higher with longer t_p_. However, the initial activity was so high that reasonable conversion higher than 50% could be achieved within 1 min, and 90% monomer could be consumed in 5 min. The activities of both catalysts dropped off monotonously over t_p_, as the concentration of the active sites was higher and the reaction conditions were more homogeneous at the early stage. While the complexed catalyst was still more active than the uncomplexed one and showed activities about 1.2–1.7 times higher under the investigated conditions. In addition, the M_w_ of the obtained PIB decreased and the MWD got broader, as the t_p_ lasted longer. This could be ascribed to chain scission as the reaction went on, and this reaction became more serious after 5 min, as the monomer conversion went up to a level higher than 90% and gave rise to monomer starvation, which would result in much broader MWD and a stronger reduction of M_n_. However, within the first 3–5 min, the M_w_ was kept high, and HPIB with M_w_ = 2–3 × 10^5^ g·mol^−1^ could be produced employing both catalysts, while the MWD was also kept relatively narrow. It indicates that t_p_ equals to 3–5 min is quite adequate to get a satisfactory monomer conversion for the synthesis of HPIB. This also implied that AlCl_3_·phenetole/TiCl_4_·H_2_O exhibited superior efficiency to the recently discovered BF_3_·EtOH/TiCl_4_·H_2_O, as the former one generally showed much higher activities than the latter one, and a much shorter t_p_ was needed for AlCl_3_·phenetole/TiCl_4_·H_2_O to achieve a sufficient monomer conversion under similar reaction conditions [27].

## 4. Conclusion

By simply compounding the high efficient AlCl_3_/phenetole for HPIB and the low efficient TiCl_4_/H_2_O for HPIB or MPIB, a novel complexed initiating system consisting of AlCl_3_·phenetole/TiCl_4_·H_2_O was successfully prepared. The contrast studies that were carried out between AlCl_3_·phenetole/TiCl_4_·H_2_O and AlCl_3_/phenetole clearly showed that a notable synergistic effect was produced in the complexed catalyst, as the activities of the complexed system were generally 1.2–3 times higher than those of the AlCl_3_/phenetole under various reaction conditions. Hence, for the complexed catalyst system, even with very low coinitiator concentration (2–5 mmol·L^−1^) and relatively high monomer concentration (ca. 4 mol·L^−1^), a satisfactory monomer conversion higher than 90% could be generally reached within 5 min. In addition, the very high activity of AlCl_3_·phenetole/TiCl_4_·H_2_O due to the synergistic effect made lower coinitiator concentration and polymerization temperature, as well as higher monomer concentration to be more favored for this complexed initiating system to produce PIBs with reasonable M_w_ and MWD. Moreover, the complexed catalyst also took advantage of AlCl_3_/phenetole in the production of HPIB, and HPIB with M_w_ = 1–3 × 10^5^ g·mol^−1^ could be synthesized under the investigated conditions. It also indicated that AlCl_3_·phenetole/TiCl_4_·H_2_O showed an enhanced competence in producing HPIB when compared with BF_3_·EtOH/TiCl_4_·H_2_O, as PIB with M_w_ = 0.8–2.2 × 10^5^ g·mol^−1^ could be produced by the latter system as a whole. Generally, the high activity as well as the simple preparation procedures of the complexed catalyst offer us a unique method for the production of HPIB with improved efficiency.

## Figures and Tables

**Table 1:** Effect of coinitiator concentration on the polymerization results ^a^.

No	Catalysts	[AlCl_3_ + TiCl_4_]	Conv.	Act. ^c^	M_w_^d^	M_n_^d^	MWD	Δ ^e^
		(mmol·L^−1^)	(%)					
1	1AlCl_3_·1phenetole	0.84	0	-	-	-	-	-
2	1AlCl_3_·1phenetole/1TiCl_4_·1H_2_O	0.84	34.9	186.14	20.89	5.50	3.8	-
3	1AlCl_3_·1phenetole	1.67	16.0	42.67	35.78	14.31	2.5	1
4	1AlCl_3_·1phenetole/1TiCl_4_·1H_2_O	1.67	53.0	141.34	20.88	5.09	4.1	3.31
5	1AlCl_3_·1phenetole	2.51	40.5	72.00	28.86	7.59	3.8	1
6	1AlCl_3_·1phenetole/1TiCl_4_·1H_2_O	2.51	95.0	168.90	20.03	0.96	20.9	2.35
7	1AlCl_3_·1phenetole	3.35	100	133.34	20.42	5.24	3.9	1
8	1AlCl_3_·1phenetole/1TiCl_4_·1H_2_O	3.35	98.7	131.61	14.37	0.98	14.7	0.99
9	1AlCl_3_·1phenetole	4.19	100	106.67	12.20	0.26	46.1	-
10	1AlCl_3_·1phenetole/1TiCl_4_·1H_2_O	5.02	97.1	86.32	12.87	0.67	19.2	-
11 ^b^	1TiCl_4_·1H_2_O	50.00	71.5	4.86	6.41	1.78	3.6	-
12	1AlCl_3_·1H_2_O	3.35	59.8	79.76	21.21	3.98	5.3	-
13	1TiCl_4_·1phenetole	1.67	N.D ^f^	N.D ^f^	N.D ^f^	N.D ^f^	N.D ^f^	-
14	1AlCl_3_·1phenetole·1H_2_O	1.67	0.6	1.47	-	-	-	-
15	1TiCl_4_·1phenetole·1H_2_O	1.67	0.4	0.90	-	-	-	-
16	1AlCl_3_·2phenetole/1TiCl_4_	1.67	0.1	0.21	-	-	-	-

^a^ For each catalyst, the molar ratio of the components is equal to that of the number in front of each component. 100 mL C_2_H_2_Cl_2_, T_p_ = −60 °C, t_p_ = 30 min, [IB] = 4 mol·L^−1^; ^b^ high [TiCl_4_] was necessary to achieve reasonable polymerization rate (see Figure S3 in Supporting Information), [IB] = 2.9 mol·L^−1^; ^c^ activity, kg PIB·mol^−1^(AlCl_3_+TiCl_4_)·h^−1^; ^d^ (×10^4^ g·mol^−1^); ^e^ Δ = activity_(complexed catalyst)_/activity_(reference catalyst)_, where both catalysts contained the same coinitiator concentration. ^f^ Not detected.

**Table 2:** Effect of reaction temperature on the polymerization results ^a^.

No	Coinitiator	T_p_	Conv.	Act. ^d^	M_w_	M_n_	MWD	Δ ^e^
		(°C)	(%)		(×10^4^ g·mol^−1^)	(×10^4^ g·mol^−1^)		
1 ^b^	AlCl_3_	−60	40.5	72.07	28.86	7.59	3.8	1
2 ^c^	AlCl_3_/TiCl_4_	−60	53.0	140.91	20.88	5.09	4.1	2.96
3 ^b^	AlCl_3_	−50	61.0	108.55	22.80	5.70	4.0	1
4 ^c^	AlCl_3_/TiCl_4_	−50	88.8	236.10	13.72	4.04	3.4	2.17
5 ^b^	AlCl_3_	−40	98.0	174.40	12.70	4.54	2.8	1
6 ^c^	AlCl_3_/TiCl_4_	−40	97.2	258.43	10.03	2.51	4.0	1.48

^a^ [IB] = 4 mol·L^−1^; 100 mL C_2_H_2_Cl_2_; t_p_ = 30 min; ^b^ [AlCl_3_] = 2.51 mmol·L^−1^, [AlCl_3_]/[phenetole] = 1/1; ^c^ [AlCl_3_] = 0.84 mmol·L^−1^, [AlCl_3_]/[TiCl_4_]/[phenetole]/[H_2_O] = 1/1/1/1; ^d^ activity, kg PIB·mol^−1^(AlCl_3_ + TiCl_4_)·h^−1^; ^e^ Δ = activity_(complexed catalyst)_/activity_(reference catalyst)_, where both the catalysts reacted at the same T_p_.

**Table 3:** Effect of monomer concentration on the polymerization results ^a^.

No	Coinitiator	[IB]	Conv.	Act. ^d^	M_w_	M_n_	MWD	Δ ^e^
		(mol·L^−1^)	(%)		(×10^4^ g·mol^−1^)	(×10^4^ g·mol^−1^)		
1 ^b^	AlCl_3_	2.4	73.8	131.27	17.90	4.97	3.6	1
2 ^c^	AlCl_3_/TiCl_4_		91.7	163.10	17.90	1.24	14.4	1.24
3 ^b^	AlCl_3_	3.3	52.5	93.38	25.47	6.70	3.8	1
4 ^c^	AlCl_3_/TiCl_4_		91.1	162.03	18.47	1.00	18.6	1.74
5 ^b^	AlCl_3_	4.0	40.5	72.04	28.86	7.59	3.8	1
6 ^c^	AlCl_3_/TiCl_4_		95.0	168.97	20.03	0.96	20.9	2.35
7 ^b^	AlCl_3_	4.6	36.4	64.74	27.23	5.79	4.7	1
8 ^c^	AlCl_3_/TiCl_4_		57.6	102.45	21.22	5.18	4.1	1.58
9 ^b^	AlCl_3_	5.1	15.4	27.39	30.30	11.22	2.7	1
10 ^c^	AlCl_3_/TiCl_4_		24.1	42.87	31.58	13.73	2.3	1.56

^a^ 100 mL C_2_H_2_Cl_2_; T_p_ = −60 °C; t_p_ = 30 min; ^b^ [AlCl_3_] = 2.51 mmol·L^−1^, [AlCl_3_]/[phenetole] = 1/1; ^c^ [AlCl_3_] = 1.26 mmol·L^−1^, [AlCl_3_]/[TiCl_4_]/[phenetole]/[H_2_O] = 1/1/1/1; ^d^ activity, kg PIB·mol^−1^(AlCl_3_ + TiCl_4_)·h^−1^; ^e^ Δ = activity_(complexed catalyst)_/activity_(reference catalyst)_, where both the catalysts reacted at the same [IB].

**Table 4:** Effect of polymerization time on the polymerization results ^a^.

No	Coinitiator	t_p_	Conv.	Act. ^d^	M_w_	M_n_	MWD	Δ ^e^
		(min)	(%)		(×10^4^ g·mol^−1^)	(×10^4^ g·mol^−1^)		
1	AlCl_3_^b^	1.0	68.8	2200.29	27.21	6.80	4.0	1.00
2	AlCl_3_/TiCl_4_^c^		51.4	2744.06	33.52	7.62	4.4	1.25
3	AlCl_3_^b^	3.0	77.7	1242.46	21.21	4.42	4.8	1.00
4	AlCl_3_/TiCl_4_^c^		63.7	1700.36	34.43	13.24	2.6	1.37
5	AlCl_3_^b^	5.0	92.3	590.37	11.97	0.44	27.2	1.00
6	AlCl_3_/TiCl_4_^c^		93.2	995.12	22.79	3.17	7.2	1.69
7	AlCl_3_^b^	10.0	95.8	306.38	11.03	0.53	20.8	1.00
8	AlCl_3_/TiCl_4_^c^		90.1	481.02	23.60	4.37	5.4	1.57
9	AlCl_3_^b^	30.0	100.0	106.67	12.20	0.26	46.1	1.00
10	AlCl_3_/TiCl_4_^c^		95.0	168.90	20.03	0.96	20.9	1.58

^a^ [IB] = 4 mol·L^−1^; 100 mL C_2_H_2_Cl_2_; T_p_ = −60 °C; ^b^ [AlCl_3_] = 4.19 mmol·L^−1^, [AlCl_3_]/[phenetole] = 1/1; ^c^ [AlCl_3_] = 1.26 mmol·L^−1^, [AlCl_3_]/[TiCl_4_]/[phenetole]/[H_2_O] = 1/1/1/1; ^d^ activity, kg PIB·mol^−1^(AlCl_3_ + TiCl_4_)·h^−1^; ^e^ Δ = activity_(complexed catalyst)_/activity_(reference catalyst)_, where the same t_p_ lasted for the both initiating systems.

## Supplementary Materials

The following are available online at https://www.mdpi.com/2073-4360/11/12/2121/s1, Figure S1: The effect of solvent polarity on TiCl_4_/H_2_O for IB polymerization ([IB] = 2.9 mol·L^−1^; [H_2_O] = 20 mmol·L^−1^; [TiCl_4_] = 30 mmol·L^−1^; t_p_=30 min; T_p_ = −60 °C.); Figure S2: The effect of T_p_ on TiCl_4_/H_2_O for IB polymerization ([IB] = 2.9 mol·L^−1^; [H_2_O] = 20 mmol·L^−1^; [TiCl_4_] = 30 mmol·L^−1^; 60 mL C_2_H_2_Cl_2_;40 mL n-hexane; t_p_ = 30 min); Figure S3: The effect of [H_2_O] and [TiCl_4_] on IB polymerization ((a) [TiCl_4_] = 50 mmol·L^−1^; (b) [H_2_O] = 40 mmol·L^−1^; (c) [TiCl_4_] = 4.56 mmol·L^−1^; Other conditions: [IB] = 2.9 mol·L^−1^; 60 mL C_2_H_2_Cl_2_; 40 mL n-hexane; t_p_ = 30 min; T_p_ = −60 °C.); Figure S4: The effect of monomer concentration on TiCl_4_/H_2_O for IB polymerization ([H_2_O] = 30 mmol·L^−1^; [TiCl_4_] = 20 mmol·L^−1^; 60 mL C_2_H_2_Cl_2_; 40 mL n-hexane; t_p_ = 30 min; T_p_ = −60 °C); Figure S5: The effect of solvent polarity on monomer conversion with AlCl_3_/phenetole initiating system ([IB] = 4 mol·L^−^^1^; V_dichloromethane_ + V_n-hexane_ = 100 mL; T_p_ = −60 °C; t_p_ = 30 min; [AlCl_3_]/[phenetole] = 1/1).
