# Supplementary material for: A Complexed Initiating System AlCl3·Phenetole/TiCl4·H2O with Dominant Synergistic Effect for Efficient Synthesis of High Molecular Weight Polyisobutylene
Source: Polymers (Basel). 2019 Dec 17;11(12):2121. doi: 10.3390/polym11122121 (PMC6960821; doi:10.3390/polym11122121)
Supplement: Supplementary file 1 [file polymers-11-02121-s001.pdf]

polymers

*Supporting Information*

# A Complexed Initiating System $AlCl_3$·Phenetole/$TiCl_4$·$H_2O$ with Dominant Synergistic Effect for Efficient Synthesis of High Molecular Weight Polyisobutylene

**Yulong Jin, Liang Chen, Xing Guo, Linfeng Xu, Zhihua Zhu, Zhen Liu, Ruihua Cheng and Boping Liu***

College of Materials and Energy, South China Agricultural University, Wushan Road 483, Guangzhou 510630, China

E-mail: boping@scau.edu.cn

State Key Laboratory of Chemical Engineering, East China University of Science and Technology, Meilong Road 130, Shanghai 200237, China

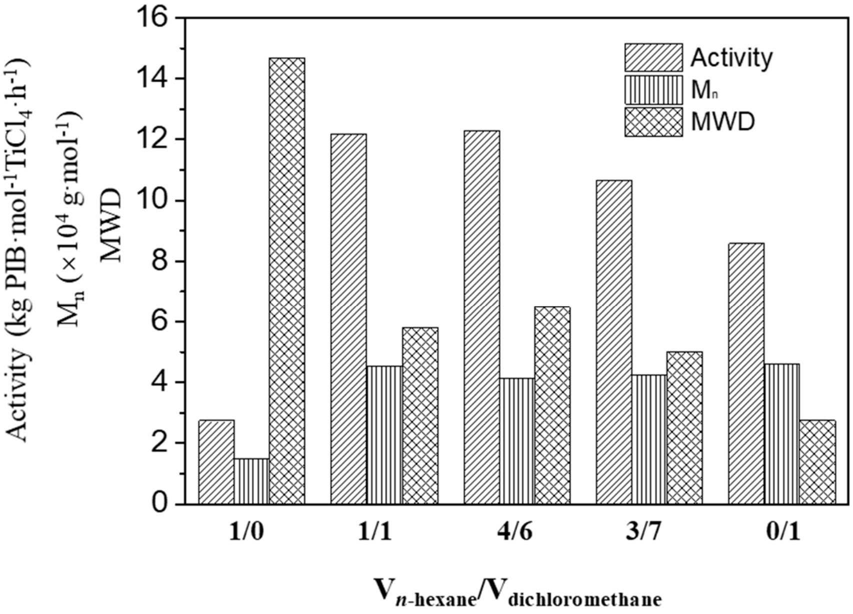


**Figure S1.** The effect of solvent polarity on $TiCl_4$/$H_2O$ for IB polymerization ([IB] = 2.9 mol·$L^{-1}$; [$H_2O$] = 20 mmol·$L^{-1}$; [$TiCl_4$] = 30 mmol·$L^{-1}$; $t_p$=30 min; $T_p$ = −60 °C.).

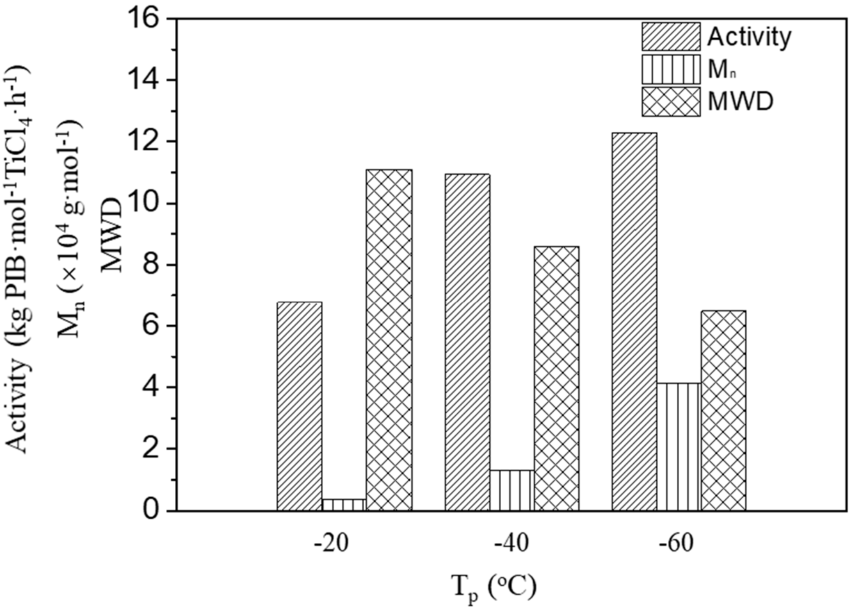


**Figure S2.** The effect of $T_p$ on $TiCl_4/H_2O$ for IB polymerization ([IB] = 2.9 mol·L$^{-1}$; [$H_2O$] = 20 mmol·L$^{-1}$; [$TiCl_4$] = 30 mmol·L$^{-1}$; 60 mL $C_2H_2Cl_2$;40 mL n-hexane; $t_p$ = 30 min)

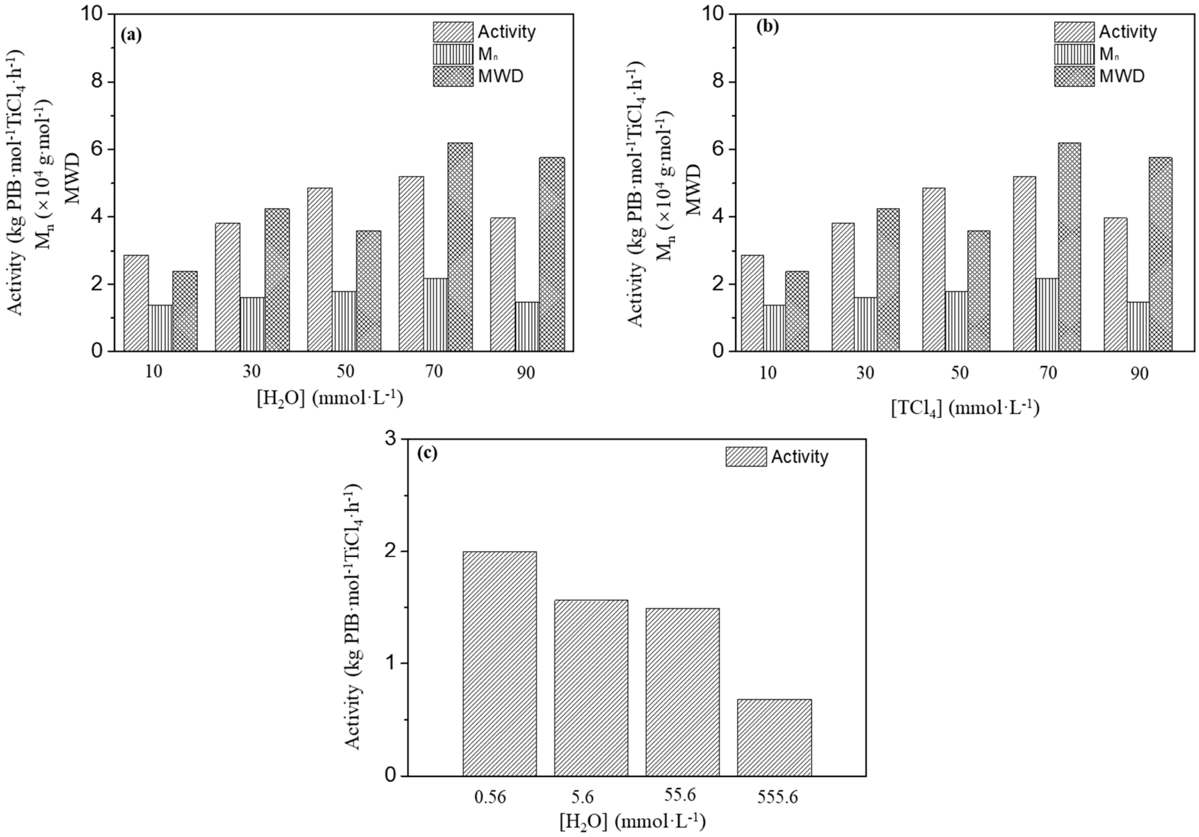


**Figure S3.** The effect of [$H_2O$] and [$TiCl_4$] on IB polymerization ((**a**) [$TiCl_4$] = 50 mmol·L$^{-1}$; (**b**) [$H_2O$] = 40 mmol·L$^{-1}$; (**c**) [$TiCl_4$] = 4.56 mmol·L$^{-1}$; Other conditions: [IB] = 2.9 mol·L$^{-1}$; 60 mL $C_2H_2Cl_2$; 40 mL n-hexane; $t_p$ = 30 min; $T_p$ = −60 °C.).

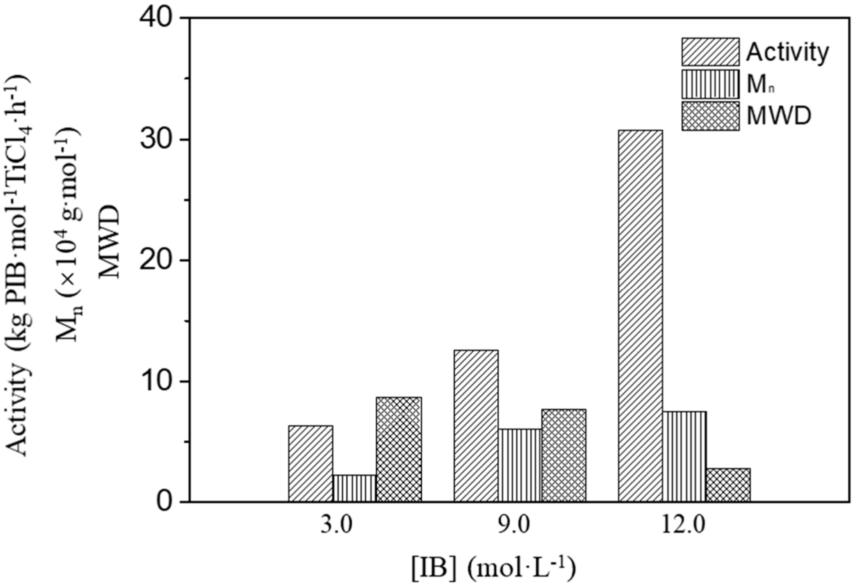


**Figure S4.** The effect of monomer concentration on $TiCl_4/H_2O$ for IB polymerization ($[H_2O]$ = 30 mmol·$L^{-1}$; $[TiCl_4]$ = 20 mmol·$L^{-1}$; 60 mL $C_2H_2Cl_2$; 40 mL n-hexane; $t_p$ = 30 min; $T_p$ = −60 °C).

The effect of various reaction conditions on the polymerization behaviors of $TiCl_4/H_2O$ were investigated as well (see **Figure S1-S4**). The results indicated that $TiCl_4/H_2O$ was good at producing high or medium molecular weight polyisobutylene with $M_n$=1~6 × $10^4$ g·$mol^{-1}$ and the MWD of the polymer was relatively broad. In addition, this initiating system showed low efficiency, and activity≈10 kg PIB·$mol^{-1}TiCl_4$·$h^{-1}$ could be observed in most cases, thus a much higher catalyst concentration was needed to achieve a reasonable monomer conversion. This was also in accordance with the reported work that the dimer of $TiCl_4$ was the active coinitiator for cationic polymerization, and a relatively high concentration of $TiCl_4$ was needed for achieving reasonable polymerization rate [S1].

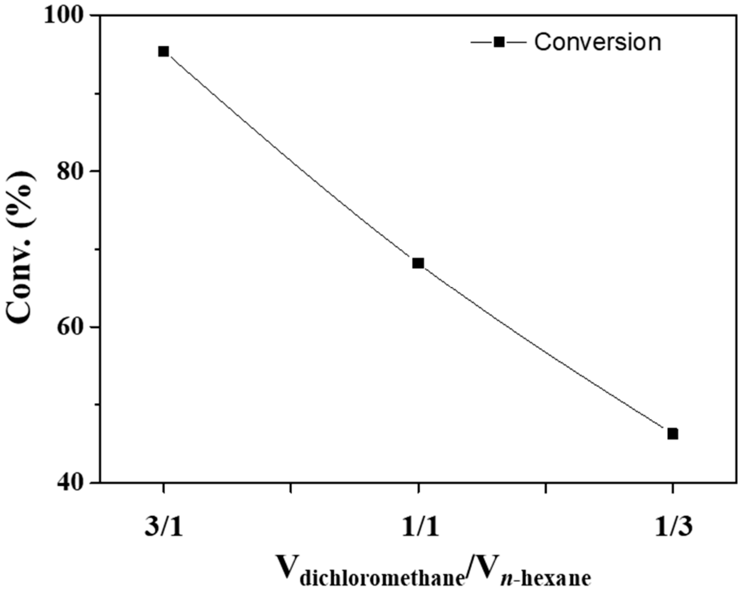


**Figure S5.** The effect of solvent polarity on monomer conversion with $AlCl_3$/phenetole initiating system ([IB] = 4 mol·$L^{-1}$; $V_{dichloromethane}$ + $V_{n\text{-}hexane}$ = 100 mL; $T_p$ = −60 °C; $t_p$ = 30 min; $[AlCl_3]$/[phenetole] = 1/1.*).*

According to **Figure S5**, a relatively stronger polarity of the solvent was more favored by the $AlCl_3$/phenetole initiating system to achieve high monomer conversion, as more polar conditions could possibly help to produce more active ion pairs for IB polymerization [S2].

## References

[S1] M. Tawada, R. Faust. *Macromolecules* **2005,** *38*, 4989.

[S2] L. Sipos, P. De, R. Faust. *Macromolecules* **2003,** *36*, 8282.

© 2020 by the authors. Submitted for possible open access publication under the terms and conditions of the Creative Commons Attribution (CC BY) license (http://creativecommons.org/licenses/by/4.0/).
